# Supplementary material for: Seminoma and Embryonal Carcinoma Footprints Identified by Analysis of Integrated Genome-Wide Epigenetic and Expression Profiles of Germ Cell Cancer Cell Lines
Source: PLoS One. 2014 Jun 2;9(6):e98330. doi: 10.1371/journal.pone.0098330 (PMC4041891; doi:10.1371/journal.pone.0098330)
Supplement: File S1 — ZIP file containing DMRforPairs output for significant regions. Please start from the html files. (ZIP) [file pone.0098330.s008.zip › figures/9415.pdf]

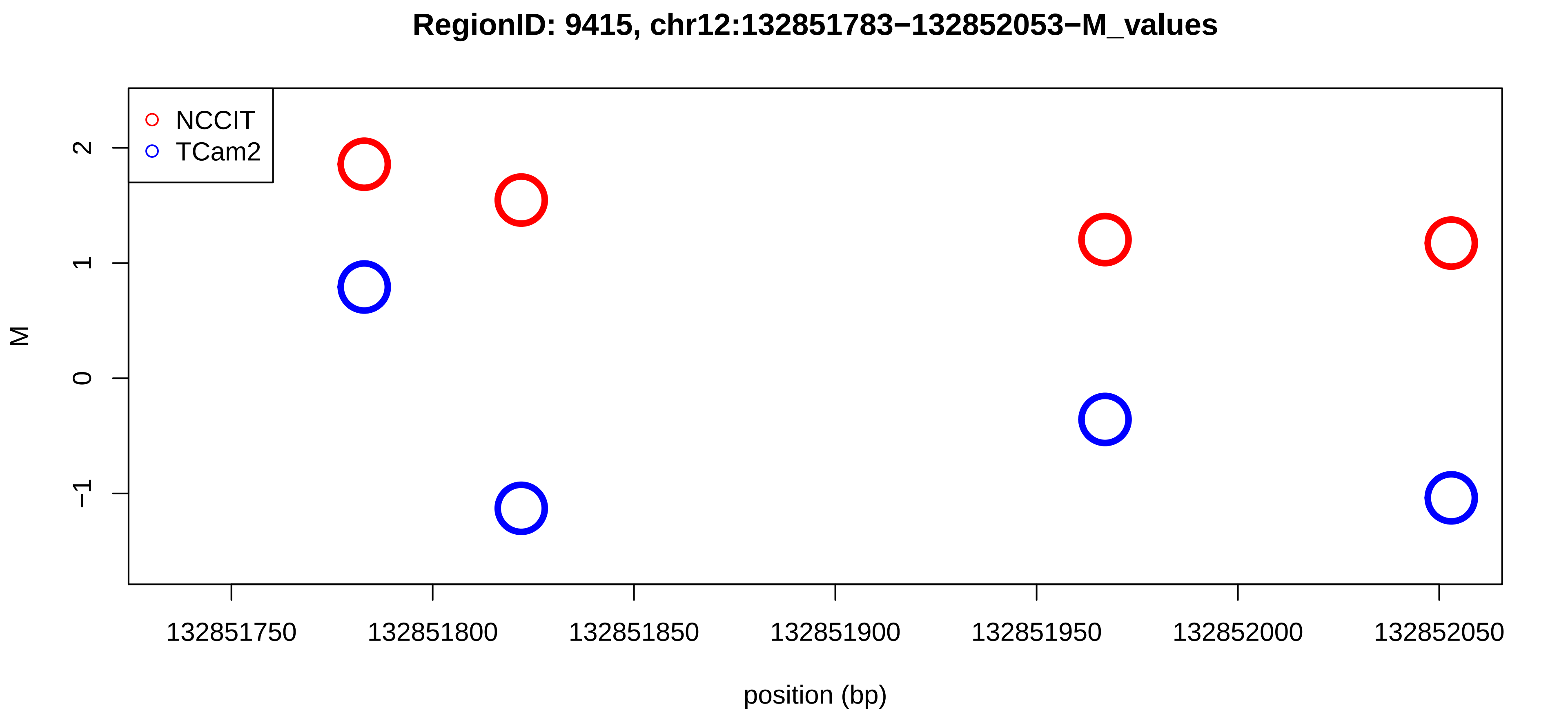
RegionID: 9415, chr12:132851783−132852053−M_values
NCCIT
TCam2
M
2
1
0
−1
132851750
132851800
132851850
132851900
132851950
132852000
132852050
position (bp)

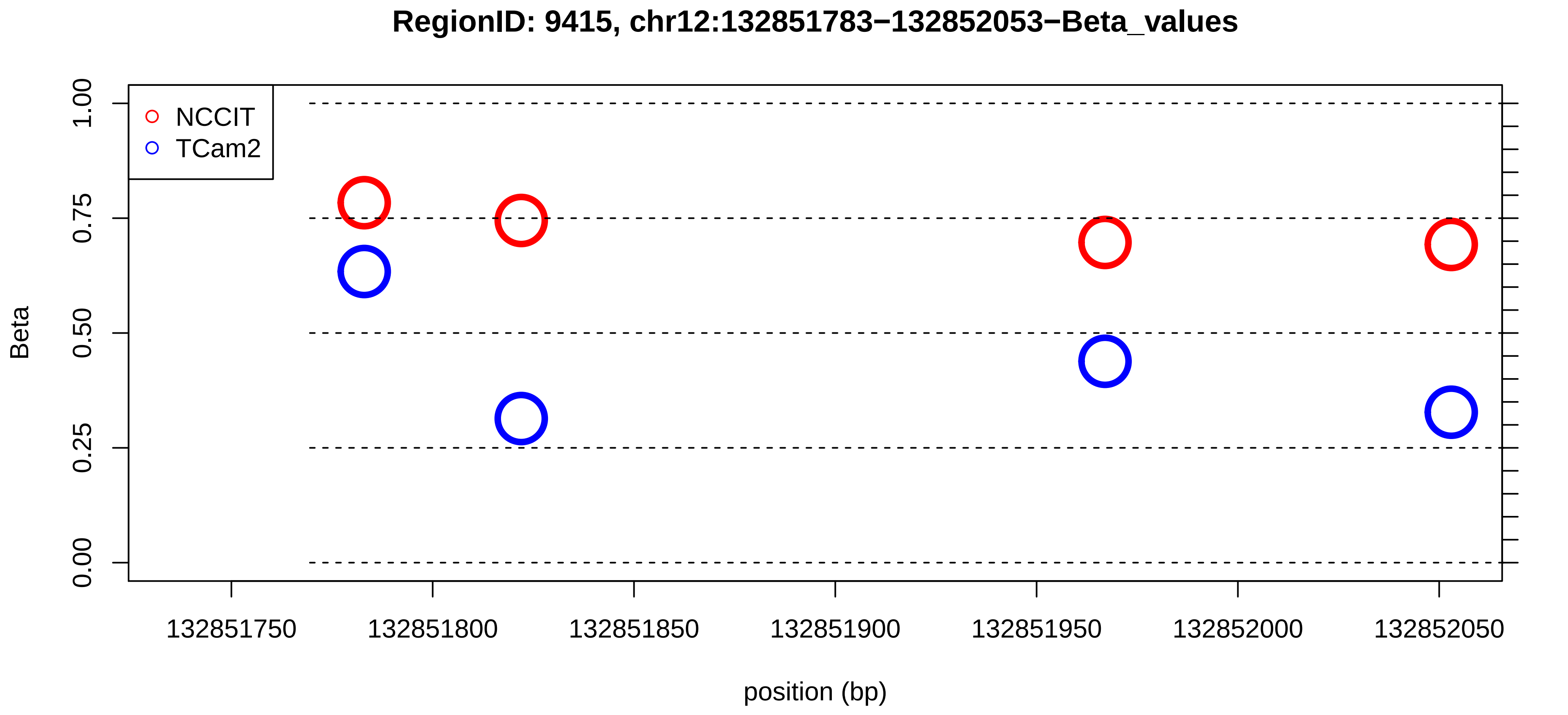
RegionID: 9415, chr12:132851783−132852053−Beta_values
NCCIT
TCam2
Beta
1.00
0.75
0.50
0.25
0.00
132851750
132851800
132851850
132851900
132851950
132852000
132852050
position (bp)
